# Feasibility and diagnostic reliability of quantitative flow ratio in the assessment of non-culprit lesions in acute coronary syndrome

**DOI:** 10.1007/s10554-021-02195-2

**Published:** 2021-03-02

**Authors:** Aslihan Erbay, Lisa Penzel, Youssef S. Abdelwahed, Jens Klotsche, Anne-Sophie Schatz, Julia Steiner, Arash Haghikia, Ulf Landmesser, Barbara E. Stähli, David M. Leistner

**Affiliations:** 1 Department of Cardiology, Charité – University Medicine Berlin, Campus Benjamin Franklin, Hindenburgdamm 30, 12203 Berlin, Germany; 2 DZHK (German Centre for Cardiovascular Research), Partner Site Berlin, Berlin, Germany; 3 German Rheumatism Research Centre Berlin, Berlin, Germany; 4 Institute for Epidemiology and Health Care Economics, Charité – University Medicine Berlin, Berlin, Germany; 5 Berlin Institute of Health (BIH), Berlin, Germany; 6 Department of Cardiology, University Heart Centre, University Hospital Zurich, Zurich, Switzerland

**Keywords:** Acute coronary syndrome, Fractional flow reserve, Quantitative flow ratio, Percutaneous coronary intervention

## Abstract

Several studies have demonstrated the feasibility and safety of hemodynamic assessment of non-culprit coronary arteries in setting of acute coronary syndromes (ACS) using fractional flow reserve (FFR) measurements. Quantitative flow ratio (QFR), recently introduced as angiography-based fast FFR computation, has been validated with good agreement and diagnostic performance with FFR in chronic coronary syndromes. The aim of this study was to assess the feasibility and diagnostic reliability of QFR assessment during primary PCI. A total of 321 patients with ACS and multivessel disease, who underwent primary PCI and were planned for staged PCI of at least one non-culprit lesion were enrolled in the analysis. Within this patient cohort, serial post-hoc QFR analyses of 513 non-culprit vessels were performed. The median time interval between primary and staged PCI was 49 [42–58] days. QFR in non-culprit coronary arteries did not change between acute and staged measurements (0.86 vs 0.87, p = 0.114), with strong correlation (r = 0.94, p ≤ 0.001) and good agreement (mean difference -0.008, 95%CI -0.013–0.003) between measurements. Importantly, QFR as assessed at index procedure had sensitivity of 95.02%, specificity of 93.59% and diagnostic accuracy of 94.15% in prediction of QFR ≤ 0.80 at the time of staged PCI. The present study for the first time confirmed the feasibility and diagnostic accuracy of non-culprit coronary artery QFR during index procedure for ACS. These results support QFR as valuable tool in patients with ACS to detect further hemodynamic relevant lesions with excellent diagnostic performance and therefore to guide further revascularisation therapy.

## Introduction

Multivessel coronary artery disease is encountered in about half of patients with acute coronary syndromes (ACS) [1–3] and is associated with an increased risk for adverse events [2–4]. Benefits of complete over culprit vessel only revascularization have been demonstrated in several studies [5, 6]. Invasive pressure-derived fractional flow reserve (FFR) has been established as gold standard for functional lesion assessment in chronic coronary syndromes (CCS) [7–9] and proves supportive results for non-culprit lesion interrogation in ACS patients [10, 11]. Resting indices such as instantaneous wave-free ratio (iFR) have also shown reasonable correlations with FFR-assessment and advantages in clinical outcome [12–14]. In patients with ACS, there is some concern that microvascular dysfunction in a highly prothrombotic and inflammatory setting could prevent reliable functional assessment of non-culprit coronary lesions [15]. However, several studies have proven the feasibility and safety of FFR measurements for non-culprit coronary arteries during ACS considering follow-up measurements as a reference [14, 16, 17]. Similarly, serial measurements of the index of microcirculatory resistance (IMR) demonstrated no significant difference between immediate and follow-up assessment [17].

Angiography-derived quantitative flow ratio (QFR) was introduced as fast FFR computation without the need for pressure wire advancement or the hyperemia induction. Large prospective trials have validated and the feasibility and diagnostic performance of QFR in comparison to FFR and resting indices [18–21]. However, data on the validity of QFR in setting of ACS are scarce.

Therefore, the aim of this study was to assess the feasibility and diagnostic reliability of QFR in non-culprit vessels of ACS patients.

## Methods

### Study population

**Fig. 1:**
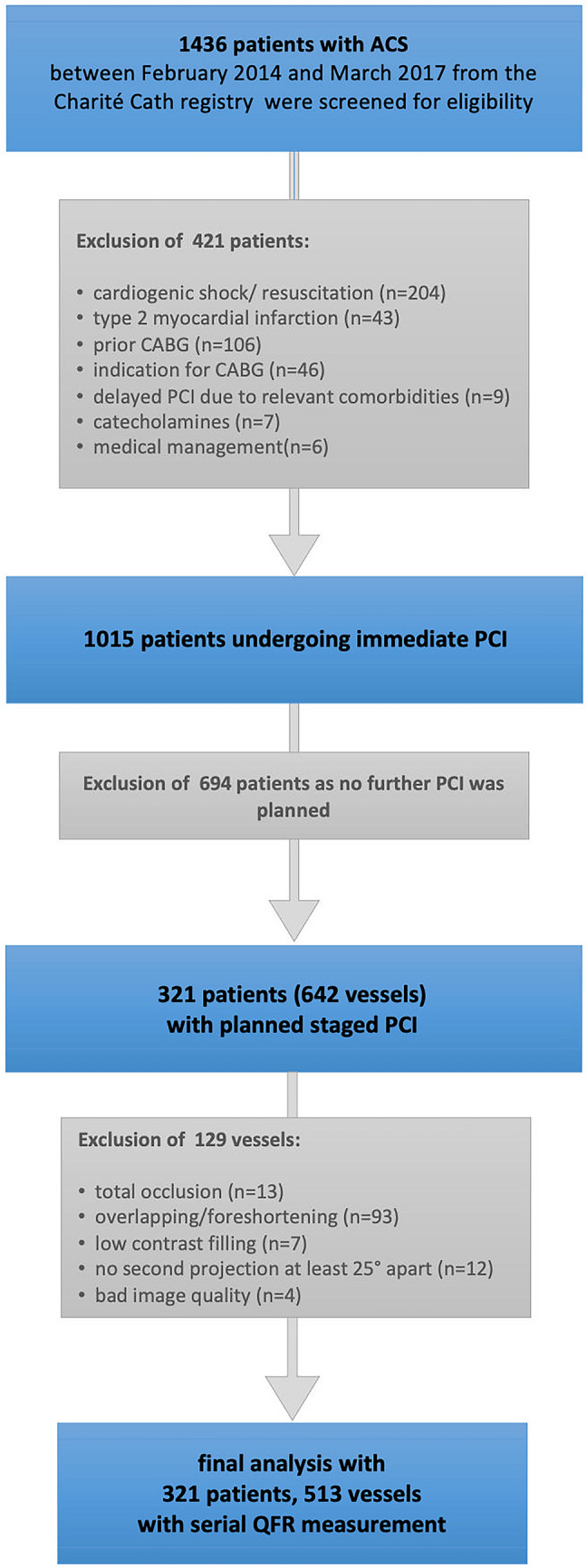
Study flow chart

A total of 1,436 patients from the Charité Cath registry, who underwent PCI for ACS between February 2014 and March 2017 at the Department of Cardiology, Charité – University Medicine Berlin, Germany, were screened for inclusion in the study. The final analysis included a total of 321 patients with planned staged PCI within six months of at least one non-culprit lesion based on visual lesion estimation of > 70% diameter stenosis on coronary angiogram at time of index ACS event (Fig. 1).

Exclusion criteria comprised ACS complicated by cardiogenic shock or resuscitation, Type 2 myocardial infarction, prior coronary artery bypass grafting (CABG), indication for CABG, atrial fibrillation, bifurcation lesions with medina classification 1–1-1, ostial lesions and angiographic characteristics precluding high-quality QFR analysis such as suboptimal angiographic image quality, lack of two projections at least 25° apart, suboptimal contrast filling, vessel overlap, or vessel foreshortening. Further, non-culprit arteries with chronic total occlusions or those undergoing immediate PCI during the index ACS procedure were excluded (Fig. 1). The registry comprised baseline characteristics, laboratory data, procedural data for the index ACS event as well as the staged procedure. All patients received evidence-based medical management and were treated with PCI according to current guidelines for myocardial revascularization and ACS [22–24].

The study was performed in accordance with the principles of the Declaration of Helsinki and local law and regulations. Ethical approval of the institutional review board was obtained.

### Quantitative flow ratio (QFR) analysis

**Fig. 2:**
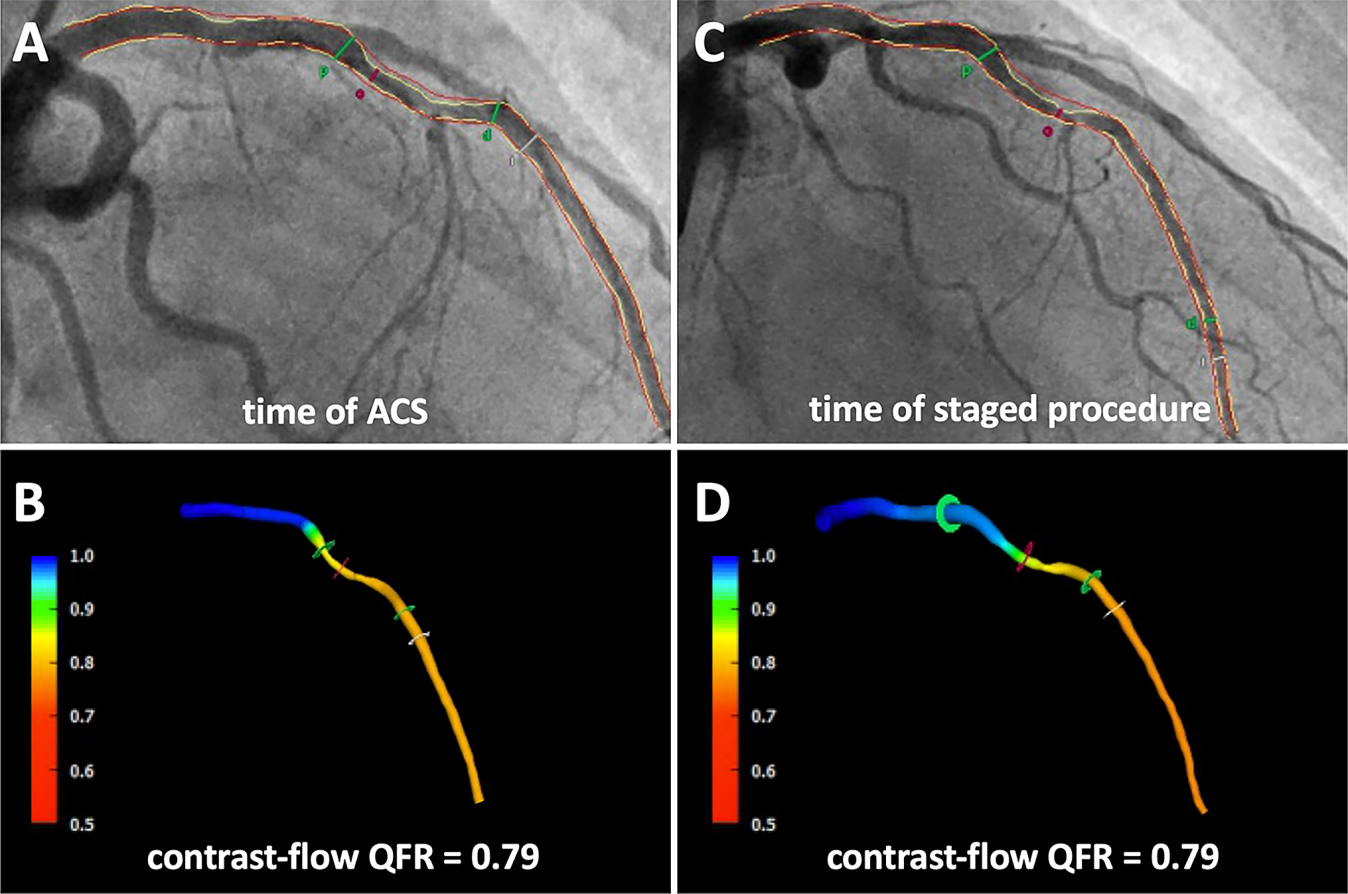
Representative case with coronary angiogram and QFR measurement of a non-culprit lesion in the medial left anterior descending coronary artery (LAD) at the time of index ACS event (**a**, **b**) and staged procedure (**c**, **d**)

Functional assessment of non-culprit coronary arteries was analysed by two certified investigators performing post-hoc three-dimensional quantitative coronary angiography (3D-QCA) and QFR analyses at the institution’s imaging core laboratory. As previously described [18, 19, 25], two angiographic projections after administration of nitroglycerine at least 25° apart with less vessel overlap and good contrast filling in the end-diastolic frames were chosen for high quality QFR analyses. After optimization of automatically detected vessel contours, the 3D vessel reconstruction was performed. In a final step, frame counting with a record of at least 12.5 frames/second allowed the calculation of contrast-flow vessel QFR (Fig. 2). The QFR investigators did not perform QFR of index and staged angiogram subsequently and were blinded to other QFR results.

### Statistical analysis

As descriptive measures, categorical variables were presented as numbers and percentages.

All continuous variables were tested for normality of distribution by the Shapiro-Wilks test and presented as mean ± standard deviation (SD) or median and interquartile range (IQR) as appropriate. Baseline characteristics were analysed at the patient level (n = 321), lesion characteristics and functional indices at the vessel level (n = 513), respectively. Acute and staged measurements of QFR and anatomic indices were compared by the Wilcoxon signed-rank test. The Spearman’s rank correlation coefficient specified the correlation between QFR at time of ACS index event and staged procedure. The agreement between serial QFR measurements was determined by Bland–Altman analysis. Sensitivity, specificity, positive predictive value (PPV), negative predictive value (NPV), positive likelihood ratio (+ LR), negative likelihood ratio (− LR), and diagnostic accuracy for predicting a hemodynamically significant coronary lesion as defined by a QFR ≤ 0.80 at the time of staged procedure were assessed. The cut-off values of 0.80 for QFR [18, 19], > 50% for diameter stenosis [18, 25], and ≥ 58% for area stenosis [26] were based on prior studies. A two-sided p-value of < 0.05 was considered statistically significant. All analyses were performed using IBM-SPSS version 26 (IBM Corp., Chicago, IL, USA).

## Results

### Baseline characteristics

Baseline patient and lesion characteristics are presented in Table 1. Median age was 66 [58—76] years, 27.1% were women. A total of 162 (50.5%) and 159 (49.5%) patients presented with ST segment elevation ACS (STE-ACS) and non-ST segment elevation ACS (NSTE-ACS), respectively. Within the study cohort, cardiovascular risk factors were frequently present such as hypertension in 94.7% or diabetes in 23% of patients. The median time interval between index and staged coronary angiography was 49 [42–58] days. Out of the analysed non-culprit vessels, 165 (32.2%) were left anterior descending arteries (LAD), 216 (42.1%) left circumflex arteries (LCX) or dominant obtuse marginal branches (OM), and 132 (25.7%) right coronary arteries (RCA).

**Table 1:** Baseline patient characteristics

At the patient level (n = 321)	
Type of ACS	
STE-ACS	162 (50.5)
NSTE-ACS	159 (49.5)
Age (years)	66 [58–76]
Male gender	234 (72.9)
Medical history	
Diabetes mellitus	67 (20.9)
Hypertension	304 (94.7)
Dyslipidemia	173 (53.9)
Prior PCI	42 (13.1)
Prior MI	31 (9.7)
Extent of CAD two-vessel-disease	158 (49.2)
Three-vessel-disease	163 (50.8)
Maximum CK level (IU/litre)	804 [328.50–1845.50]
LVEF (%)	53 [45–60]

Values are given as median and interquartile range or counts and percentages

*ACS* acute coronary syndrome, *STE-ACS* ST segment elevated acute coronary syndrome, *NSTE-ACS* non-ST segment elevated acute coronary syndrome, *PCI* percutaneous coronary intervention, *MI* myocardial infarction, *CAD* coronary artery disease, *CK* creatine kinase, *LVEF* left ventricular ejection fraction

### Functional assessment of non-culprit vessels

In 521 coronary arteries, serial 3D-QCA and QFR analyses were performed. Minimum lumen diameter of the analysed non-culprit vessels was 1.4 [1.0–1.8] mm at baseline and 1.3 [1.0–1.8] mm at follow-up, while the diameter stenosis was 46.6 [36.2–57.3] % and 45.3 [35.2–56.1]%, respectively. Hemodynamic significance defined as QFR ≤ 0.80 was observed in 211 (41.1%) non-culprit vessels at time of ACS and in 201 (39.2%) vessels at staged procedure.

### Feasibility and diagnostic reliability of QFR in ACS

**Fig. 3:**
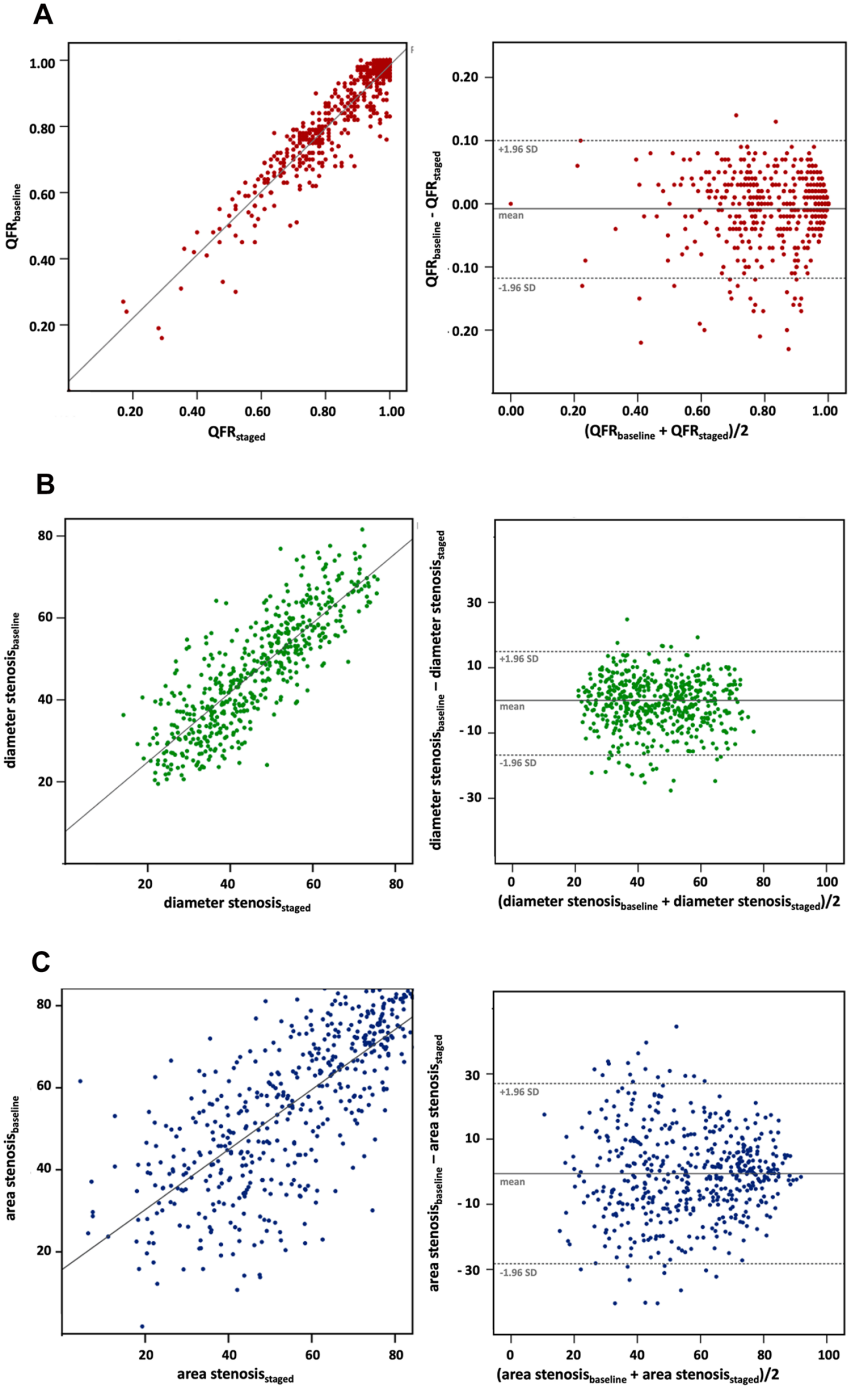
Correlation and agreement between serial QFR assessment and anatomic indices at baseline and staged procedure. **a** Correlation and Bland Altman plot between QFR at baseline and staged procedures. **b** Correlation and Bland Altman plot between diameter stenosis at baseline and staged procedures. **c** Correlation and Bland Altman plot between area stenosis at baseline and staged procedures

Contrast-flow vessel QFR in non-culprit coronary arteries was comparable between acute and staged measurements (0.86 [0.74–0.97] vs. 0.87 [0.75–0.97], p = 0.11) (Table 2). Strong correlation (r = 0.94 (95% CI 0.93–0.95), p ≤ 0.001) and good agreement (mean difference -0.008, 95% CI -0.013–0.003) between serial QFR measurements were observed (Fig. 3). Importantly, QFR as assessed at index procedure had high sensitivity (95.0%), specificity (93.6%), PPV (90.5%), NPV (96.7%), and diagnostic accuracy (94.2%) in predicting QFR of ≤ 0.80 at time of staged procedure (Table 3). Corresponding values for anatomic indices are given in Tables 2, 3.

**Table 2:** Vessel characteristics at baseline and follow-up

At the vessel level (n = 513)	
Localization LAD	165 (32.2)
LCX/OM/IM	216 (42.1)
RCA	132 (25.7)

	Baseline	Follow-Up	p-value
Fixed-flow vessel QFR	0.86 [0.74–0.97]	0.87 [0.75–0.97]	0.309
Contrast-flow vessel QFR	0.86 [0.74–0.97]	0.88 [0.75–0.97]	0.114
Lesion length (mm)	17.50 [10.80–26.55]	16.80 [10.05–26.70]	0.487
Reference diameter (mm)	2.70 [2.30–3.00]	2.60 [2.20–3.00]	0.008
Diameter stenosis (%)	46.60 [36.20–57.25]	45.30 [35.20–56.05]	0.098
Area stenosis (%)	58.20 [41.30–72.55]	57.40 [41.15–72.95]	0.266
Minimum lumen diameter (mm)	1.40 [1.00–1.80]	1.30 [1.00–1.80]	0.330
Plaque volume (mm^3^)	29.20 [14.20–56.45]	24.20 [11.35–54.65]	0.140

Values are given as median and interquartile range or counts and percentages

*LAD* left anterior descending coronary artery, *LCX* left circumflex coronary artery, *OM* obtuse marginal branch, *IM* intermediate coronary artery, *RCA* right coronary artery, *QFR* quantitative flow ratio

**Table 3:** Diagnostic performance of contrast-flow vessel QFR, diameter stenosis and area stenosis with staged indices as reference

n = 513 vessels	QFR ≤ 0.80	Diameter stenosis ≥ 50%	Area stenosis ≥ 58%
Sensitivity	95.02 (91.04–97.59)	85.99 (80.50–90.41)	84.25 (79.18–88.50)
Specificity	93.59 (90.27–96.04)	86.27 (81.90–89.93)	81.08 (75.77–85.66)
Accuracy	94.15 (91.76–96.02)	86.16 (82.87–89.03)	82.65 (79.09–85.83)
Positive predictive value	90.52 (86.19–93.59)	80.91 (76.09–84.95)	81.37 (77.14–84.96)
Negative predictive value	96.69 (94.10–98.16)	90.10 (86.62–92.75)	84.00 (79.70–87.53)
Positive likelihood ratio	14.82 (9.69–22.68)	6.27 (4.71–8.34)	4.45 (3.44–5.76)
Negative likelihood ratio	0.05 (0.03–0.10)	0.16 (0.12–0.23)	0.19 (0.15–0.26)
Mean difference ± SD	− 0.01 ± 0.05	− 0.91 ± 7.9	− 0.61 ± 13.82

Serial QFR measurements revealed 26 non-culprit lesions (5.1%) whose QFR result at time of ACS were different at staged procedure. Out of these 26 non-culprit lesions, 18 lesions with hemodynamic relevance at time of ACS had no longer functional significance at staged procedure (QFR 0.76 [0.71–0.78] at baseline vs. QFR 0.85 [0.82–0.88] at follow-up, p < 0.001). Otherwise, QFR revealed hemodynamic relevant stenoses at staged procedure in 8 cases which were not considered significant at time of ACS procedure (QFR 0.84 [0.83–0.87] at baseline vs. QFR 0.78 [0.76–0.80] at follow-up, p < 0.05).

## Discussion

The present study investigated for the first time the feasibility and diagnostic reliability of angiography-derived functional assessment of non-culprit vessels in ACS by post-hoc serial QFR measurements in a reasonable study population of ACS patients. Our findings demonstrate that hemodynamic assessment by QFR in the acute phase of ACS represents a robust diagnostic tool for non-culprit lesion assessment. In only 5.1% of patients, discordance in QFR values between the acute phase and the staged procedure were observed. Importantly, angiography-based QFR outperformed anatomic indices as assessed by 3D-QCA.

### Hemodynamic assessment of non-culprit lesions in ACS

There is no doubt for primary PCI as the first-line therapy in patients with ACS [22–24]. Given that multivessel disease is present in about a half of ACS patients [1–3], the optimal treatment of non-culprit lesions is of substantial interest. The superiority of complete coronary revascularization over culprit-only PCI was proven in several studies [5, 6, 10, 11]. An FFR-guided revascularization strategy further bears the advantage of achieving improved outcomes with a lower number of subsequent revascularizations and consecutivelylower health care costs [27].

### QFR as an alternative modality to invasive physiological assessment

Quantitative flow ratio represents an angiography-based functional tool for the identification of ischemia-causing lesions by fast FFR computation. Limiting aspects of invasive FFR measurement such as additional instruments, prolongation of procedural duration, and an increased procedural risk by FFR-wire advancement and induction of hyperemia, along with increased procedural costs, can be completely avoided. Recently, it was demonstrated that the expenditure of time for QFR assessment was significantly less than the time to complete FFR measurement [19]. Thereby, QFR poses an attractive and well-suited tool for fast functional lesion assessment in the setting of ACS.

The present study now extends the findings regarding feasible and reliable functional lesion assessment in ACS from wire- and adenosine-based measurements to angiography-based functional lesion interrogation by demonstrating the high reproducibility and excellent diagnostic accuracy of QFR in a large cohort of patients with ACS and serial QFR measurements. Importantly, QFR outperformed anatomic indices such as diameter stenosis and bears the potential of a more accurate assessment of lesion severity. Our findings are in line with previous studies including patients with ACS and undergoing staged FFR evaluation [28–31]. The present results for serial QFR assessment underline the reliable performance of QFR in setting of ACS and provides a good basis for following outcome studies.

## Limitations

A few limitations need to be considered. The present study was a retrospective and not previously specified analysis. As a result, 3D-QCA and QFR analyses were performed from available coronary angiographies which were not optimally obtained according to QFR acquisition guide and led to an exclusion of 129 (20.1%) vessels due to suboptimal angiography or vessel overlap at acute or staged procedures. Nevertheless, the study represents a realistic impression of every-day clinical routine and a high-quality QFR analysis could be performed in 321 patients with 513 non-culprit vessels, corresponding to 79.9% of all eligible vessels. In addition, the reference was determined as QFR results from staged angiogram and no wire-based assessment by FFR as the actual gold standard in functional assessment of coronary lesions. Since the good correlation and diagnostic performance compared to FFR has been shown many times [18–21], this study focused on serial QFR measurement. The strong correlations observed in this study further support the wide applicability and feasibility of QFR in everyday clinical practice.

## Conclusion

The present study demonstrates the feasibility and high diagnostic reliability of QFR assessment in non-culprit coronary arteries during ACS. These results support QFR as valuable tool for the detection of hemodynamic relevant lesions in patients with ACS. The prognostic impact of QFR and its role for PCI guidance in ACS has to be evaluated in future prospective randomized clinical outcome trials.

## Data Availability

Data and material are available on request.

## Abbreviations

3D-QCA: Three-dimensional quantitative coronary angiography
ACS: Acute coronary syndrome
CABG: Coronary artery bypass grafting
CCS: Chronic coronary syndrome
FFR: Fractional flow reserve
iFR: Instantaneous wave-free ratio
LVEF: Left ventricular ejection fraction
NSTE-ACS: Non-ST segment elevation acute coronary syndrome
PCI: Percutaneous coronary intervention
QFR: Quantitative flow ratio
STE-ACS: ST segment elevation acute coronary syndrome
